# Viral polymerase inhibitors T-705 and T-1105 are potential inhibitors of Zika virus replication

**DOI:** 10.1007/s00705-017-3436-8

**Published:** 2017-06-08

**Authors:** Lei Cai, Yajie Sun, Yabin Song, Likun Xu, Zhuchun Bei, Dongna Zhang, Yuanyuan Dou, Hongquan Wang

**Affiliations:** State Key Laboratory of Pathogen and Biosecurity, Beijing Institute of Microbiology and Epidemiology, Academy of Military Medical Science, Beijing, 100071 China

**Keywords:** Zika virus (ZIKV), Broad-spectrum antiviral drugs, T-705, T-1105

## Abstract

Since 2015, 69 countries and territories have reported evidence of vector-borne Zika virus (ZIKV) transmission. Currently, there are no effective licensed vaccines or drugs available for the treatment or prevention of ZIKV infection. We tested a series of compounds for their ability to inhibit ZIKV replication in cell culture. The compounds in T-705 (favipiravir) and T-1105 were found to have antiviral activity, suggesting that these compounds are promising candidates for further development as specific antiviral drugs against ZIKV.

**Electronic supplementary material:**

The online version of this article (doi:10.1007/s00705-017-3436-8) contains supplementary material, which is available to authorized users.

## Introduction

Zika virus (ZIKV) is a member of the viral family *Flaviviridae* and the genus *Flavivirus* [1]. It is named after the Zika Forest of Uganda, where the virus was first isolated in 1947 [2]. ZIKV has reemerged in recent years, causing global outbreaks [3, 4]. In May 2015, a ZIKV outbreak was first reported in Brazil, and at least 69 countries and territories have since reported evidence of vector-borne ZIKV transmission [4]. The illness is usually mild, with symptoms lasting for several days [5]. However, there is now scientific consensus that ZIKV is linked to severe fetal malformations, serious disorders of the central nervous system [6, 7], and Guillain-Barré syndrome [4, 8], which has caused widespread concern. On February 1, 2016, the World Health Organization declared that the recent association of ZIKV infection with clusters of microcephaly and other neurological disorders constitutes a Public Health Emergency of International Concern. Therefore, increasing numbers of scientists are working on anti-ZIKV drugs or vaccines, and some progress has been made [9–11].

The development of highly effective, broad-spectrum antiviral agents is the major objective shared by the fields of virology and pharmaceutics. In this study, to identify a promising candidate drug to treat this disease, we evaluated ribavirin, CMX001 (brincidofovir), T-705 (favipiravir), and T-1105 in cell culture to assess their ability to inhibit ZIKV infection. Ribavirin was first reported in 1972 as a broad-spectrum antiviral drug that is active against a variety of RNA and DNA viruses *in vitro* and *in vivo* [12, 13]. CMX001 is an experimental antiviral agent infection for the treatment of cytomegalovirus, adenovirus, smallpox virus, and Ebola virus infections [14–16]. T-705, a nucleoside analogue that was first reported in 2002 [17], exerts potent broad-spectrum antiviral effects against many viruses, including influenza A, B, and C viruses [18, 19], human and avian viruses [12], and even Ebola virus (in 2014) [20]. This drug is also active against a wide variety of unrelated RNA viruses (reviewed by Furuta and coworkers) [21]. T-1105, a structural analogue of T-705, has been reported to exert broad-spectrum antiviral effects against some RNA virus, including foot-and-mouth disease virus and bovine viral diarrhea virus [21].

In the present study, the anti-ZIKV activity of a series of T-705 analogues substituted with several functional groups, including alkyl, ester, and aryl glycoside moieties, was evaluated. T-705 and T-1105 were found to have antiviral activity, suggesting that these two compounds are promising candidates for the further development of specific antiviral drugs against ZIKV.

**Fig. 1:**
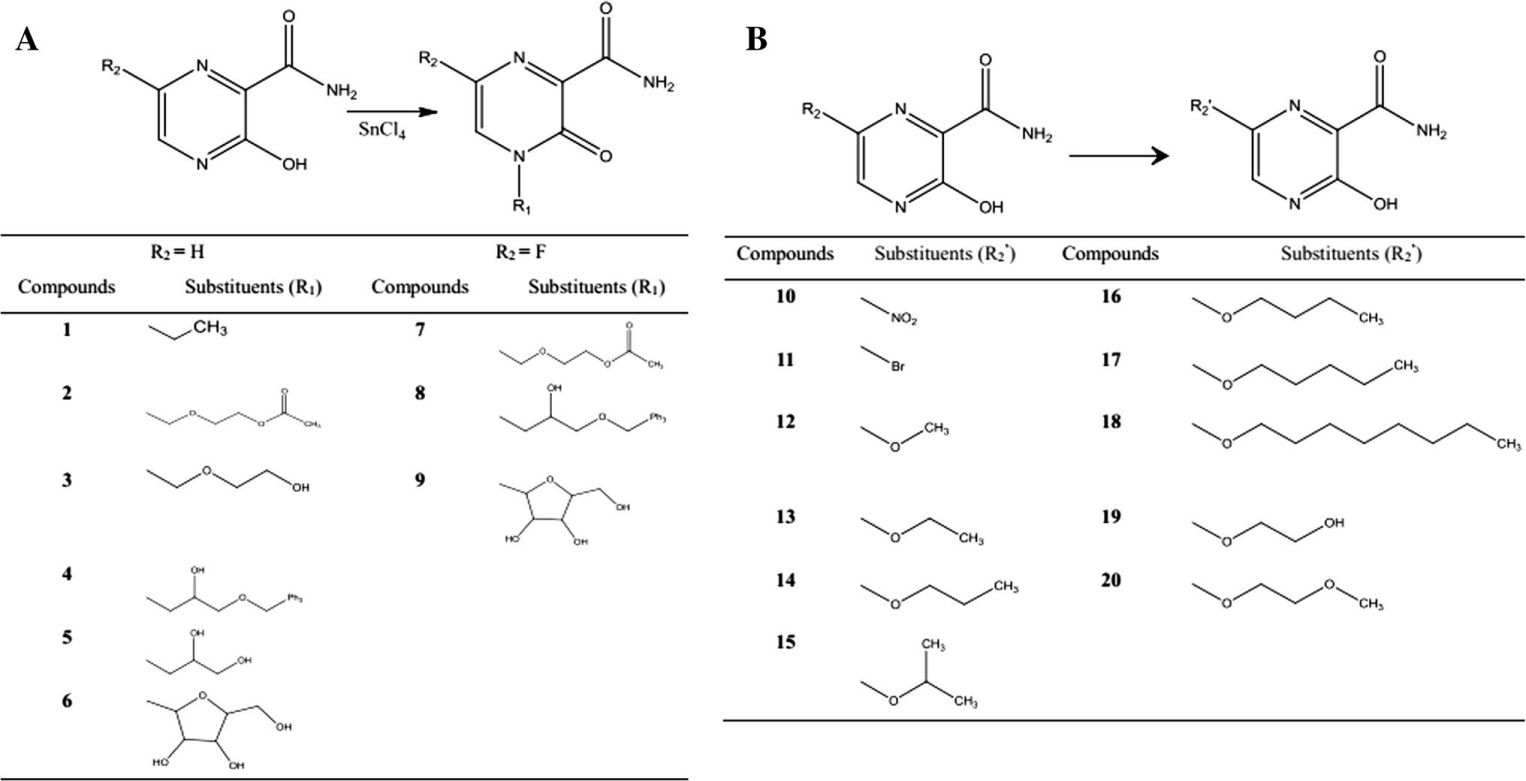
The synthetic route to the target compounds **A.** compounds (1–9). **B**. compounds 10–20

A series of T-705 analogues (compounds 1–20) substituted with several functional groups, including alkyl, ester, and aryl glycoside moieties, were synthesized by the following methods (Fig. 1).

## 4-ethyl-3-oxo-3,4-dihydropyrazine-2-carboxamide (1)

T-1105 (1.39 g, 10.0 mmol) was dissolved in hexamethyldisilazane (10 ml), heated to 130 °C, and stirred for 6 h. After it was cooled to room temperature and concentrated, dry acetonitrile (15 ml) and ethyl chloride (0.78 g, 12.1 mmol) were added, and the mixture was cooled to 0 °C. Dry SnCl_4_ (1.2 ml, 10.2 mmol) was added slowly, and the reaction mixture was then heated to room temperature and stirred for 5 h. The reaction was terminated with saturated NaHCO_3_ solution, and the pH was adjusted to neutral before the solution was extracted with ethyl acetate. The organic phase was dried over Na_2_SO_4_, filtered, and concentrated to a sticky residue. The residue was purified by silica column chromatography to afford compound 1 (1.02 g, 6.1 mmol, 61.1% yield).

## 2-((3-carbamoyl-2-oxopyrazin-1(2H)-yl) methoxy) ethyl acetate (2)

Compound 2 was synthesized using a method similar to that described above for compound 1, with 23.8% yield.

## 4-((2-hydroxyethoxy) methyl)-3-oxo-3,4-dihydropyrazine-2-carboxamide (3)

Compound 2 (2.55 g, 10.0 mmol) was dissolved with 1 M sodium methoxide (1.8 ml) and stirred for 20 min. The activated cation exchange resin (4 g) was added, The suspension was was stirred for 30 min, the pH was adjusted to neutral with dilute hydrochloric acid, and the sample was filtered. The solvent was evaporated to afford compound 3 (1.33 g, 5.2 mmol, 62.4% yield)

## 4-(2-hydroxy-3-(trityloxy) propyl)-3-oxo-3,4-dihydropyrazine-2-carboxamide (4)

T1105 (1.39 g, 10.0 mmol) was dissolved in hexamethyldisilazane (30 ml), heated to 130 °C, and stirred for 6 h. After it was cooled to room temperature and concentrated, dry dimethylformamide (60 ml), s-(-)-2-((trityloxy) methyl) oxirane (10.01 g, 32.2 mmol), and K_2_CO_3_ (0.82 g, 5.9 mmol) were added, and the reaction mixture was stirred at 90 °C for 40 h. The reaction was terminated with water (70 ml) and the material was extracted with ethyl acetate. The organic phase was collected and dried over NaSO_4_, filtered, and concentrated to a sticky residue. The residue was purified by silica column chromatography to afford 4 (3.24 g, 7.1 mmol, 22.4% yield).

## 4-(2,3-dihydroxypropyl)-3-oxo-3,4-dihydropyrazine-2-carboxamide(5)

Compound 4 (2.02 g, 4.5 mmol) was dissolved in 80% HCOOH (20 ml) and stirred at room temperature for 30 min. The reaction mixture was extracted with water and dichloromethane. The aqueous phase was collected and concentrated to give a sticky residue. The product was isolated by silica gel column chromatography and dried to give compound 5 (0.54 g, 2.25 mmol, 53.3% yield).

## 4-(3,4-dihydroxy-5-(hydroxymethyl)tetrahydrofuran-2-yl)-3-oxo-3,4-dihydropyrazine-2-carboxamide (6)

T-1105 (7.01 g, 50.4 mmol) was dissolved in hexamethyldisilazane (50 ml), heated to 130 °C, and stirred for 6 h. After it was cooled to room temperature and concentrated, tetraacetyl ribose (3.90 g, 12.2 mmol) was dissolved in acetonitrile (75 ml), and the mixture was cooled to 0 °C. Dry SnCl_4_ (5.75 ml, 49.1 mmol) was added slowly dropwise and the mixture was stirred at room temperature overnight. The reaction was terminated with saturated NaHCO_3_ solution, the pH was adjusted to neutral, and the solution was extracted with ethyl acetate. The organic phase was dried over Na_2_SO_4_, filtered, and concentrated to a sticky residue. The product was isolated on a silica gel column and dried to give intermediate (5.45 g, 13.7 mmol). The intermediate (5.45 g, 13.7 mmol) was dissolved in methyl alcohol (60 ml), and the mixture was cooled to 0 °C. 5 M sodium methoxide (6.11 g) was added, and the mixture was stirred for 30 min. The pH was adjusted to neutral with acetic acid, and the material was filtered and concentrated to a sticky residue. The residue was purified by silica column chromatography to afford 6 (1.36 g, 5.0 mmol, 50.2% yield).

## 2-((3-carbamoyl-5-fluoro-2-oxopyrazin-1(2H)-yl) methoxy) ethyl acetate (7)

Compound 7 was synthesized using a method similar to that described above for compound 2, with 19.6% yield.

## 6-fluoro-4-(2-hydroxy-3-(trityloxy)propyl)-3-oxo-3,4-dihydropyrazine-2-carboxamide (8)

Compound 8 was synthesized using a method similar to that described above for compound 4, with 16.3% yield.

## 4-(3,4-dihydroxy-5-(hydroxymethyl)tetrahydrofuran-2-yl)-6-fluoro-3-oxo-3,4-dihydropyrazine-2-carboxamide (9)

Compound 9 was synthesized using a method similar to that described above for compound 6, with 69.9% yield.

## 6-nitropyrazine-3-hydroxy-2-carboxamide (10)

T-705 (1.57 g, 10.0 mmol) was dissolved in 60% nitric acid (9 ml) and cooled to 0 °C. SOCl_2_ (1.2 ml, 16.9 mmol) was added slowly and stirred at room temperature for 2 h. The material was stored at 4 °C overnight and filtered to afford compound 10 (1.23 g, 6.7 mmol, 66.8% yield).

## 6-bromo-3-hydroxypyrazine-2-carboxamide (11)

T-1105 (1.51 g, 10.8 mmol) and pyridine (1.5 ml) were added to dimethylformamide (4 ml) to form an emulsion. The reaction was heated to 80 °C and liquid bromine (0.7 ml, 13.7 mmol) was added slowly. The reaction mixture was stirred at room temperature for 2 h, and the reaction was terminated with water (6 ml), cooled, and filtered. Then, the filter cake was washed with a small amount of water to afford compound 11 (1.15 g, 5.3 mmol, 48.8% yield).

## 3-hydroxy-6-methoxypyrazine-2-carboxamide (12)

T-705 (1.57 g, 10.0 mmol) was dissolved in methyl alcohol (5 ml, 64.5 mmol). SOCl_2_ (1.2 ml, 16.9 mmol) was added slowly, and the mixture was heated to 40 °C and stirred for 2 h. It was then cooled to room temperature and filtered to afford compound 12 (0.98 g, 5.8 mmol, 57.9% yield).

## 6-ethoxy-3-hydroxypyrazine-2-carboxamide (13)

Compound 13 was synthesized using a method similar to that described above for compound 12, with 68.8% yield.

## 3-hydroxy-6-propoxypyrazine-2-carboxamide (14)

Compound 14 was synthesized using a method similar to that described above for compound 12, with 77.2% yield.

## 6-isopropoxypyrazine-3-hydroxy-2-carboxamide (15)

Compound 15 was synthesized using a method similar to that described above for compound12, with 68.0% yield.

## 6-butoxy-3-hydroxypyrazine-2-carboxamide (16)

Compound 16 was synthesized using a method similar to that described above for compound 12, with 59.2% yield.

## 3-hydroxy-6-(pentyloxy)pyrazine-2-carboxamide (17)

Compound 17 was synthesized using a method similar to that described above for compound12, with 68.4% yield.

## 3-hydroxy-6-(octyloxy)pyrazine-2-carboxamide (18)

Compound 18 was synthesized using a method similar to that described above for compound 12, with 74.1% yield.

## 3-hydroxy-6-(2-hydroxyethoxy) pyrazine-2-carboxamide (19)

Compound 19 was synthesized using a method similar to that described above for compound 12, with 76.8% yield.

## 3-hydroxy-6-(2-methoxyethoxy) pyrazine-2-carboxamide (20)

Compound 20 was synthesized using a method similar to that described above for compound 12, with 64.3% yield.

We tested a series of compounds for their ability to inhibit ZIKV replication in cell culture. In this study, Vero cells were incubated at 37 °C in Dulbecco’s modified Eagle medium (HyClone) with 10% fetal bovine serum (Gibco) [22]. The ZIKV strain SZ01 (GenBank accession number KU866423.2) was used in the experiment. It was isolated from a Chinese patient who had returned from Samoa in 2016, and it belongs to the Asian lineage [23]. For cell culture experiments, ribavirin, CMX001, T-705 and T-1105 (all >95% purity) were initially dissolved in 100% dimethyl sulfoxide (DMSO). The compounds were then diluted to the desired concentrations in 1% DMSO.

The Spearman–Karber method was used to determine the ZIKV stock titers [24]. Briefly, Vero cells (approximately 3 × 10^4^ cells/well) were seeded in the wells of a 96-well plate and incubated at 37 °C under an atmosphere of 5% CO_2_ for 24 h before infection. The cells were infected with serial dilutions of the virus from 10^−1^ to 10^−10^ (100 μl/well), incubated at 37 °C, and monitored for any cytopathic effect (CPE). The viral titer was calculated using the Spearman–Karber formula, and the results are presented as the half-maximal (50%) tissue culture infective dose (TCID_50_).

The cytotoxicity of the compounds against Vero cells was determined by monitoring the CPE. Vero cells (2.5 × 10^4^/well) were plated in the wells of a 96-well plate. After 24 h, the cell monolayer was incubated with different concentrations of the test compounds for 72 h at 37 °C under a 5% CO_2_ atmosphere in a CO_2_ incubator and then monitored for CPE. The appropriate negative and positive controls were used. A neutral red uptake assay [25] was used to assess cell viability. The half-maximal (50%) cytotoxic concentration (CC_50_) of each compound was determined from the dose-response curve.

The anti-ZIKV activities of the compounds were evaluated in Vero cells using a CPE inhibition assay [22]. Confluent Vero cells grown in 96-well microplates were infected with 68.6 TCID_50_ of the test strain and then treated with fivefold serial dilutions of the test compounds. Incubation with each concentration was performed in triplicate. The vehicle, 1% DMSO (final concentration), was used as the negative control. The CPE was monitored until it reached a level 4 in the cells of the control group. The neutral red uptake assay was used to assess cell viability. The half-maximal (50%) effective concentration (EC_50_) of each compound was determined from the dose-response curve.

This study was designed to improve the activity of the drug T-705, reduce its organ-targeting toxicity, and develop a new candidate treatment for ZIKV infection. T-705 has a simple structure, and we engineered changes at two positions. As shown in Fig. 1, the chemical structures of the target analogues included changes to the substituents on the pyrazine at two positions, 4 and 6. We selected 3-hydroxypyrazine-2-carboxamide as the lead compound, and 20 compounds were synthesized with structural modifications by introducing functional groups at the 4 and 6 positions, including alkyl, ester, and sugar moieties.

**Fig. 2:**
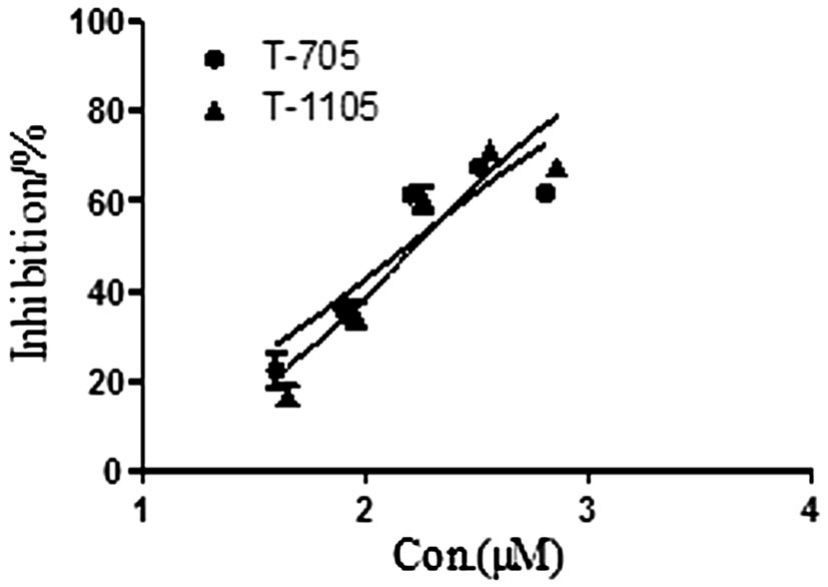
Dose-response curves showing the *in vitro* antiviral activity of T-705 and T-1105 against ZIKV strain SZ01. Prism 5.0 software was used to plot the data from parallel experiments

We evaluated the anti-ZIKV activities of these compounds against ZIKV strain SZ01 in Vero cells. The synthetic compounds had weak or no antiviral activity in the concentration range of 200–600 μM. Ribavirin was not active at concentrations below 400 μM, and CMX001 was not active at concentrations below 18 μM. As shown in Table 1, ribavirin and CMX001 had little or no effect on ZIKV-induced CPE or cell death. However, T-705 and T-1105 significantly reduced the cell death rate in the test wells compared with that in the mock-treated ZIKV-infected cells (*P* < 0.05, two-tailed Student’s *t*-test). The EC_50_ of T-705 was 110.9 ± 13.1 μM and that of T-1105 was 97.5 ± 6.8 μM. The dose-response curve results are shown in Fig. 2.

**Table 1:** Antiviral activity of compounds against ZIKV strain SZ01

Compound	CC_50_ (μM)	EC_50_ (μM)
Ribavirin	>2000	NA
CMX001	11.3 ± 0.2	NA
T-705	>3000	110.9 ± 13.1
T-1105	>4000	97.5 ± 6.8
1	>1000	NA
2	>2000	NA
3	>1000	NA
4	>500	NA
5	>2000	NA
6	>1000	NA
7	>1000	NA
8	>500	NA
9	>1000	NA
10	>4000	NA
11	>2000	NA
12	>1000	NA
13	>500	NA
14	>500	NA
15	345.4 ± 48.4	NA
16	309.5 ± 15.2	NA
17	259.9 ± 51.1	NA
18	245.4 ± 17.8	NA
19	>1000	NA
20	>1000	NA

Determined from three independent experiments

CC_50_, 50% cytotoxic concentration; EC_50_, 50% effective concentration; SD, standard deviation, NA, not applicable

The continuation of outbreaks of ZIKV infection in Southeast Asia and the Americas emphasizes the urgent need for effective antiviral drugs. Many scientists have been working on anti-ZIKV drugs [10, 11, 26, 27], including D. Růžek and co-workers [26], who suggested that 2′-C-methylated nucleosides might be promising candidates for the development of specific antiviral drugs against ZIKV. In this study, we found that T-705 and T-1105 effectively inhibited ZIKV *in vitro*.

ZIKV, like other flaviviruses, is an enveloped, single-stranded positive-sense RNA virus [28]. It has been hypothesized that ZIKV uses the RNA-dependent RNA polymerase (RdRp or the non-structural protein, NS5), together with cofactors to replicate, maintain its infection, and express its RNA genome [26–28]. T-705 is converted to the active form, ribofuranosyl triphosphate (T-705-RTP), by host cell enzymes and selectively inhibits the activity of the influenza viral RNA polymerase [15]. T-705 also inhibits several pathogenic flaviviruses, including yellow fever virus (YFV) and West Nile virus (WNV) [29, 30], which prompted the hypothesis that T-705 inhibits the polymerases of YFV and WNV. Therefore, we suggest that T-705 also acts by inhibiting the RNA polymerase of ZIKV.

A series of derivatives of T-705 were tested, and these compounds had little or no effect on ZIKV-induced CPE or cell death. Several functional groups, including alkyl, ester, and sugar moieties, were introduced into compounds 1–9 at the 4-N position. Compounds 6 and 9 are the ribosides of T-1105 and T-705, respectively. These modified molecules did not inhibit the replication of ZIKV, and we suggest two possible mechanisms to explain this failure. One possible explanation is that the side chains of the substituents altered the oil/water partition coefficients, which may have changed the cell permeability of the compounds. Another explanation may be that the effective concentrations were higher than the test concentrations. As reported by Furuta *et al*., compound 6 was effective in a hamster model of YFV infection, although it was previously shown to have only slight activity at low concentrations in cell culture [28]. In a future study, we will test the antiviral activities of these compounds at higher concentrations and *in vivo* to identify other effective compounds for the treatment of ZIKV disease. In compounds 10–20, the –F moiety was substituted with other groups, including nitro and ether groups. These side chains may have changed the charge distribution on the molecules or caused greater steric hindrance effects than F and H. Therefore, the derivatives of T-705 do not act like T-705-RTP in inhibiting the viral RNA polymerase in host cells.

In conclusion, we have demonstrated that the derivatives of T-705 had little or no effect at the tested concentrations (about 200–600 μM). However, T-705 and T-1105 displayed anti-ZIKV activity *in vitro*. In a future study, we will test the antiviral activities of the substituted compounds at higher concentrations and undertake *in vivo* experiments to identify other effective compounds for the treatment of ZIKV disease.

## Electronic supplementary material

Below is the link to the electronic supplementary material.

Supplementary material 1 (DOC 49 kb)
