# Supplementary material for: Viral polymerase inhibitors T-705 and T-1105 are potential inhibitors of Zika virus replication
Source: Arch Virol. 2017 Jun 8;162(9):2847–53. doi: 10.1007/s00705-017-3436-8 (PMC5563514; doi:10.1007/s00705-017-3436-8)
Supplement: Supplementary file 1 — Supplementary material 1 (DOC 49 kb) [file 705_2017_3436_MOESM1_ESM.doc]

1

^1^H NMR (600 MHz, DMSO-*d*_6_) δ 1.26 (t, *J* = 7.2 Hz, 3H), 3.97 (q, *J* = 7.2 Hz, 2H), 7.50 (d, *J* = 4.0 Hz, 1H), 7.70 (s, 1H), 7.93 (d, *J* = 4.1 Hz, 1H), 8.44 (s, 1H).

HRMS-ESI (*m/z*): calculated for C_7_H_9_N_3_O_2_ [2M+Na] ^+^:357.0695; measured: 357.1282.

2

^1^H NMR (600 MHz, CDCl_3_) δ 2.03 (s, 3H), 3.76 – 3.91 (m, 2H), 4.11 – 4.28 (m, 2H), 5.44 (s, 2H), 6.49 (s, 1H), 7.54 (d, *J* = 4.1 Hz, 1H), 7.73 (d, *J* = 4.1 Hz, 1H), 9.04 (s, 1H).

HRMS-ESI (*m/z*): calculated for C_10_H_15_N_3_O_5_ [M-H] -:256.0855; measured: 256.0928.

3

^1^H NMR (600 MHz, DMSO-*d*_6_) δ 3.50 (t, *J* = 5.1 Hz, 2H), 3.56 (t, *J* = 5.0 Hz, 2H), 4.71 (t, *J* = 5.4 Hz, 1H), 5.35 (s, 2H), 7.49 (d, *J* = 4.1 Hz, 1H), 7.71 (s, 1H), 7.86 (d, *J* = 4.2 Hz, 1H), 8.31 (s, 1H).

HRMS-ESI (*m/z*): calculated for C_8_H_10_N_3_O_4_ [M-H] -:212.0750; measured: 212.0677.

4

^1^H NMR (600 MHz, DMSO-*d*_6_) δ 2.92 (dd, *J* = 9.4, 6.0 Hz, 1H), 3.03 (dd, *J* = 9.4, 5.0 Hz, 1H), 3.70 (dd, *J* = 12.8, 9.0 Hz, 1H), 3.98 – 4.06 (m, 1H), 4.37 (dd, *J* = 12.8, 3.5 Hz, 1H), 5.33 (d, *J* = 5.9 Hz, 1H), 7.23 – 7.29 (m, 3H), 7.34 (t, *J* = 7.8 Hz, 6H), 7.41 (d, *J* = 7.2 Hz, 6H), 7.45 (d, *J* = 4.0 Hz, 1H), 7.69 – 7.71 (m, 1H), 7.73 (d, *J* = 4.0 Hz, 1H), 8.46 (d, *J* = 1.2 Hz, 1H).

HRMS-ESI (*m/z*): calculated for C_27_H_25_N_3_O_4_ [M-H] -:454.1845; measured: 454.1772.

[M+Na] ^+^:478.1845; measured: 478.1737.

5

^1^H NMR (600 MHz, DMSO-*d*_6_) δ 3.33 (dt, *J* = 11.4, 5.9 Hz, 1H), 3.41 (dt, *J* = 10.7, 5.3 Hz, 1H), 3.59 (dd, *J* = 12.8, 9.1 Hz, 1H), 3.77 (td, *J* = 5.6, 2.9 Hz, 1H), 4.30 (dd, *J* = 12.8, 3.2 Hz, 1H), 4.77 (t, *J* = 5.7 Hz, 1H), 5.04 (d, *J* = 5.7 Hz, 1H), 7.47 (d, *J* = 4.0 Hz, 1H), 7.68 (s, 1H), 7.75 (d, *J* = 4.1 Hz, 1H), 8.47 (s, 1H).

HRMS-ESI (*m/z*): calculated for C_8_H_12_N_3_O_4_ [M+Na] ^+^:236.0750; measured: 236.0642.

6

^1^H NMR (600 MHz, DMSO-*d*_6_) δ 3.61 – 3.67 (m, 1H), 3.77 – 3.82 (m, 1H), 3.99 – 4.03 (m, 1H), 5.08 (d, *J* = 5.4 Hz, 1H), 5.25 (t, *J* = 4.9 Hz, 1H), 5.60 (d, *J* = 4.6 Hz, 1H), 5.92 (d, *J* = 2.5 Hz, 1H), 7.53 (d, *J* = 4.3 Hz, 1H), 7.71 (s, 1H), 8.27 (d, *J* = 4.3 Hz, 1H), 8.34 (s, 1H).

HRMS-ESI (*m/z*): calculated for C_10_H_18_N_3_O_6_ [M-H] -:272.0804; measured: 272.0877.

[M+Na] ^+^:294.0804; measured: 294.0697.

7

^1^H NMR (600 MHz, CDCl_3_) δ 2.06 (t, *J* = 4.8 Hz, 3H), 3.83 – 3.87 (m, 2H), 4.24 (t, *J* = 4.8 Hz, 2H), 5.46 (s, 2H), 7.56 (s, 1H), 7.57 (s, 1H), 9.08 (s, 1H).

HRMS-ESI (*m/z*): calculated for C_10_H_12_F_1_N_3_O_5_ [M-H] -:272.0761; measured: 272.0688. [M+Na] ^+^:296.0761; measured: 296.0653.

8

^1^H NMR (600 MHz, CDCl_3_) δ 3.14 – 3.25 (m, 3H), 3.80 – 3.94 (m, 1H), 4.11 – 4.26 (m, 1H), 4.47 (dd, *J* = 13.1, 3.0 Hz, 1H), 7.22 – 7.26 (m, 6H), 7.27 – 7.31 (m, 3H), 7.38 – 7.41 (m, 6H), 7.43 (d, *J* = 6.0 Hz, 1H), 8.64 (d, *J* = 5.0 Hz, 1H), 9.16 (s, 1H).

HRMS-ESI (*m/z*): calculated for C_27_H_24_F_1_N_3_O_4_ [M-H] -:472.1751; measured: 472.1678.

[M+Na] ^+^:496.1751; measured: 496.1643.

9

^1^H NMR (600 MHz, DMSO-*d*_6_) δ 3.27 – 3.33 (m, 2H), 3.65 (dd, *J* = 12.4, 2.1 Hz, 1H), 3.86 (dd, *J* = 12.4, 2.4 Hz, 1H), 3.95 – 3.99 (m, 1H), 4.03 (d, *J* = 6.1 Hz, 1H), 5.39 – 5.48 (m, 1H), 5.74 (s, 1H), 5.84 (s, 1H), 7.89 – 7.94 (m, 1H), 8.60 (d, *J* = 5.2 Hz, 1H), 8.62 (s, 1H).

HRMS-ESI (*m/z*): calculated for C_10_H_12_F_1_N_3_O_6_ [M-H] -:288.0710; measured: 288.0637.

10

^1^H NMR (600 MHz, CDCl_3_) δ 7.26 (s, 1H), 7.58 (s, 1H), 8.49 (s, 1H), 12.48 (s, 1H).

HRMS-ESI (*m/z*): calculated for C_5_H_4_N_4_O_4_ [M-H] -:183.0233; measured: 183.0160.

11

^1^H NMR (600 MHz, CDCl_3_) δ 7.26 (s, 1H), 7.58 (s, 1H), 8.49 (s, 1H), 12.48 (s, 1H).

HRMS-ESI (*m/z*): calculated for C_5_H_4_Br_1_N_3_O_2_ [M-H] -:215.9487; measured: 215.9414.

12

^1^H NMR (600 MHz, DMSO-*d*_6_) δ 12.78 (s, 1H), 8.52 (s, 1H), 8.37 (s, 1H), 8.15 (s, 1H), 3.92 (s, 4H).

HRMS-ESI (*m/z*): calculated for C_6_H_7_N_3_O_3_ [M-H] -:168.0487; measured: 168.0415.

13

^1^H NMR (600 MHz, CDCl_3_) δ 11.88 (s, 1H), 8.10 (s, 1H), 7.41 (d, *J* = 7.7 Hz, 1H), 5.86 (s, 1H), 4.30 (q, *J* = 7.1 Hz, 2H), 1.41 (d, *J* = 7.2 Hz, 3H).

HRMS-ESI (*m/z*): calculated for C_7_H_9_N_3_O_3_ [M-H] -:182.0644; measured: 182.0571.

14

^1^H NMR (600 MHz, Chloroform-*d*) δ 11.91 (s, 1H), 8.09 (s, 1H), 7.45 (s, 1H), 6.52 (s, 1H), 4.17 (d, *J* = 6.6 Hz, 2H), 1.84 – 1.74 (m, 2H), 1.01 (d, *J* = 7.2 Hz, 3H).

HRMS-ESI (*m/z*): calculated for C_8_H_11_N_3_O_3_ [M-H] -:196.0800; measured: 196.0728.

15

^1^H NMR (600 MHz, Chloroform-*d*) δ 11.88 (s, 1H), 8.04 (s, 1H), 7.41 (s, 1H), 6.46 (s, 1H), 5.25 – 4.85 (m, 1H), 1.34 (d, *J* = 6.2 Hz, 6H).

HRMS-ESI (*m/z*): calculated for C_8_H_11_N_3_O_3_ [M-H] -:196.0800; measured: 196.0728.

16

^1^H NMR (600 MHz, Chloroform-*d*) δ 11.92 (s, 1H), 8.05 (s, 1H), 7.48 (s, 1H), 6.85 (s, 1H), 4.20 (t, *J* = 6.6 Hz, 2H), 1.75 – 1.67 (m, 2H), 1.47 – 1.38 (m, 2H), 0.92 (t, *J* = 7.4 Hz, 3H).

HRMS-ESI (*m/z*): calculated for C_9_H_13_N_3_O_3_ [M-H] -:210.0957; measured: 210.0884.

17

^1^H NMR (600 MHz, Chloroform-*d*) δ 11.89 (s, 1H), 8.08 (s, 1H), 7.44 (s, 1H), 6.48 (s, 1H), 4.21 (t, *J* = 6.6 Hz, 2H), 1.79 – 1.72 (m, 2H), 1.44 – 1.31 (m, 4H), 0.90 (d, *J* = 7.2 Hz, 3H).

HRMS-ESI (*m/z*): calculated for C_10_H_15_N_3_O_3_ [M-H] -:224.1113; measured: 224.1041.

18

^1^H NMR (600 MHz, Chloroform-*d*) δ11.88 (s, 1H), 8.10 (s, 1H), 7.41 (s, 1H), 5.90 (s, 1H), 4.21 (t, *J* = 6.6 Hz, 2H), 1.92 – 1.61 (m, 2H), 1.54 – 1.13 (m, 10H), 0.87 (t, *J* = 7.0 Hz, 3H).

HRMS-ESI (*m/z*): calculated for C_8_H_11_N_3_O_3_ [M-H] -:266.1573; measured: 266.1510.

19

^1^H NMR (600 MHz, DMSO-*d*_6_) δ 3.70 (q, *J* = 5.1 Hz, 2H), 4.36 (dd, *J* = 5.7, 4.3 Hz, 2H), 4.81 (t, *J* = 5.5 Hz, 1H), 8.14 (s, 1H), 8.35 (s, 1H), 8.53 (s, 1H), 12.79 (s, 1H).

HRMS-ESI (*m/z*): calculated for C_7_H_9_N_3_O_4_ [M-H] -:198.0593; measured: 198.0520.

20

^1^H NMR (600 MHz, Chloroform-*d*) δ 11.91 (s, 1H), 8.17 (s, 1H), 7.38 (s, 1H), 5.82 (s, 1H), 4.46 – 4.23 (m, 2H), 3.74 (t, *J* = 4.2 Hz, 2H), 3.43 (s, 3H).

HRMS-ESI (*m/z*): calculated for C_8_H_11_N_3_O_4_ [M-H] -:212.07500; measured: 212.0677.
